# Effective control of postoperative recurrence of pregnancy-related gastric cancer using anti-PD-1 as a monotherapy: a case report

**DOI:** 10.3389/fonc.2024.1321149

**Published:** 2024-05-10

**Authors:** Xu Liu, Xiaoqi Li, Chunchao Zhu, Linhua Ji

**Affiliations:** Department of Gastrointestinal Surgery, Ren Ji Hospital, School of Medicine, Shanghai Jiao Tong University, Shanghai, China

**Keywords:** pregnancy-related gastric cancer, tumor recurrence, immunotherapy, anti-PD-1, monotherapy

## Abstract

Pregnancy-related gastric cancer is characterized by a refractory nature and poor prognosis; few gastric cancer cases during pregnancy achieved acceptable outcomes by using anti-PD-1 as a monotherapy. A 32-year-old pregnant female patient was admitted to the emergency department of the obstetrics and gynecology department and eventually diagnosed with gastric cancer. Radical surgery for gastric cancer was conducted after the termination of pregnancy. At 1-year postoperative follow-up, tumor recurrence was revealed. This patient has achieved a decrease in tumor burden after receiving anti-PD-1 as a monotherapy. This case documents tumor response to PD-1 monotherapy in pregnancy-related gastric cancer and highlights the potential for future use in specific clinical scenarios.

## Introduction

The process of diagnosis and treatment of gastric cancer during pregnancy is quite challenging, which unavoidably presents patients with conflicting choices of individualized treatment and continued childbirth. This group of gastric cancer patients is characterized by a refractory nature and dismal prognosis (1–3).

As for the treatment options, immunotherapy, especially with immune checkpoint blockades, has changed the direction of cancer care. Programmed cell death protein-1 (PD-1) blockage is the most widely used method for immune checkpoint inhibition (4). Nevertheless, only some particular gastric cancer patients have achieved promising results after receiving anti-PD-1 therapy. There are very few gastric cancer patients during pregnancy who achieved satisfactory outcomes by using immunotherapy alone (5).

This was a case of a patient with gastric cancer during pregnancy who underwent radical gastric cancer surgery after elective induction of labor, yet postoperative tumor recurrence occurred. Nevertheless, the patient refused chemotherapy and was treated with anti-PD-1 therapy alone. Intriguingly, the recurrent lesion was found to have continued to shrink during the subsequent follow-up. The aim of this study is to provide experience and protocols for the comprehensive treatment of patients with pregnancy-related gastric cancer.

We present a case of a pregnancy-related gastric cancer patient who achieved promising results with immunotherapy alone. The study including the human participant was reviewed and approved by the Research Ethics Committee of Ren Ji Hospital, School of Medicine, Shanghai Jiao Tong University and was carried out in accordance with the ethical standards of the World Medical Association’s Declaration of Helsinki. The patient provided written informed consent.

## Case presentation

A 32-year-old female patient was admitted to the department of Emergency Obstetrics and Gynecology, Ren Ji Hospital, Shanghai Jiao Tong University (Shanghai, China) with an emergency admission of 11 h for hematemesis with epigastric pain while at 21 + 4 weeks of pregnancy on August 10, 2018. The patient had regular menstruation, the age of menarche was 12 years old, and the menstrual cycle was 5–7/30 with moderate volume and no dysmenorrhea. Her fertility history was 0–0-0–0, LMP: 2018–03-18, and EDC: 2018–12-30. Early pregnancy ultrasound verified the gestational weeks, 4 months of menopause, and felt fetal movement. The patient experienced severe vomiting on May 10, 2018 with pink foam in the vomitus and no concomitant symptoms such as abdominal pain, diarrhea, or melena. The patient regarded it as morning sickness and did not pay enough attention to it. Despite that, the vomiting gradually worsened until the patient was unable to eat normally. On August 1, 2018, she was presented to one of the maternal and child health hospitals and was later recommended to be referred to Renji Hospital. Family and social history was non-contributory and unremarkable. Her past history included *in vitro* fertilization–embryo transfer.

Upon physical examination, the vital signs were stable, with a mild anemic appearance, a mid to lower abdominal bulge, and mild epigastric pain. The patient was admitted with black vomitus. The abdominal circumference was 94 cm and the fundal height was 30 cm. There were no contractions, and the fetal heart rate was 149 bpm.

**Figure 1:**
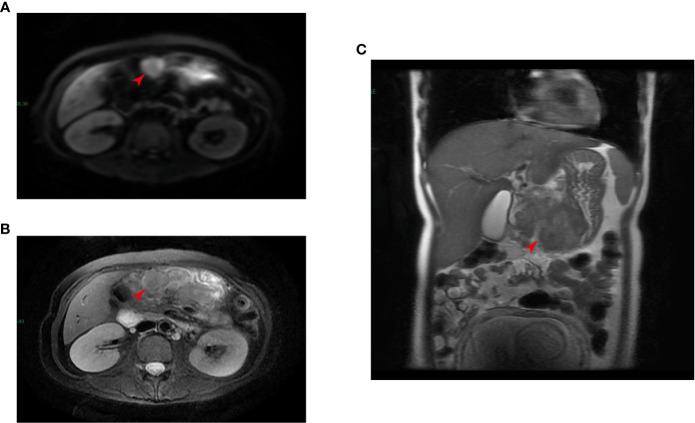
Magnetic resonance imaging (MRI) detected a tumor in the gastric sinus (as indicated by the red arrow, intersecting surface **(A, B)**; coronal position **(C)**).

At auxiliary examination, the emergency gynecological ultrasound revealed as follows: singleton cephalic position, fetal heart rate and fetal movement: visible, growth meridian: 51–188-158–33. The emergency ancillary tests revealed as follows: Hb: 95 g/L; gastric fluid occult blood: positive, fecal occult blood: negative; AFP: 90.20 ng/mL (normal range: 0–25 ng/mL); CA 724: 172.10 U/ml (normal range: 0–6 U/mL), CYFRA (21–1): 11.48 ng/ml (normal range: 0–3.3 ng/mL), and CA242: 23.5 U/ml (normal range: 0–20 U/mL). The upper abdomen enhanced MRI showed a space-occupying lesion of the gastric sinus, suspicious lymph node enlargement on the lateral side of the greater curvature of the gastric sinus, and a scan of the pregnant uterus (**Figures 1A–C**). Electron fiberoptic gastroscopy revealed a large, ulcerated lesion of the gastric sinus, involving the four walls and the gastric angle, and the lesser curvature of the gastric body (**Supplementary Figure S1**). The gastroscopic diagnosis was malignant ulcer of the gastric sinus with incomplete obstruction, and the pathological diagnosis was poorly differentiated adenocarcinoma. Moreover, the immunohistochemistry results indicated Her-2(-); the tumor was classified as cTNM:cT4N+M0. Based on the medical history and auxiliary examination results detailed above, the following diagnosis has been made: gastric adenocarcinoma, singleton pregnancy, and moderate anemia.

After conducting MDT discussions in Renji hospital, radical surgery for gastric cancer was recommended after termination of pregnancy. She was then induced in our Obstetrics and Gynecology Department (22 + 1 weeks gestation), and the placenta-fetal membranes were sent for pathology for the presence of tumor cells. The pathology suggested “placenta-fetal membranes” and placenta membrane tissue with degeneration; no tumor tissue was seen. On August 30, 2018, radical surgery for gastric cancer was performed (major distal gastrectomy with remnant gastrojejunostomy RY anastomosis, anterior colon, and gastric D2 lymph node dissection). Intraoperatively, the gross appearance of the resected specimen was a tumor located in the anterior wall of the gastric body and the gastric sinus, with a size of about 4 × 5 cm in diameter, stiff, infiltrative ulcerative type, with a central deep concave ulcer, with the tumor breaking through the plasma membrane layer and invading part of the transverse colonic mesentery and the pancreatic capsule, and perigastric nos. 6, 7, 8, 9, and 12a lymph nodes were accessible and enlarged. No obvious metastatic lesions were found in the abdominal and pelvic cavities.

**Figure 2:**
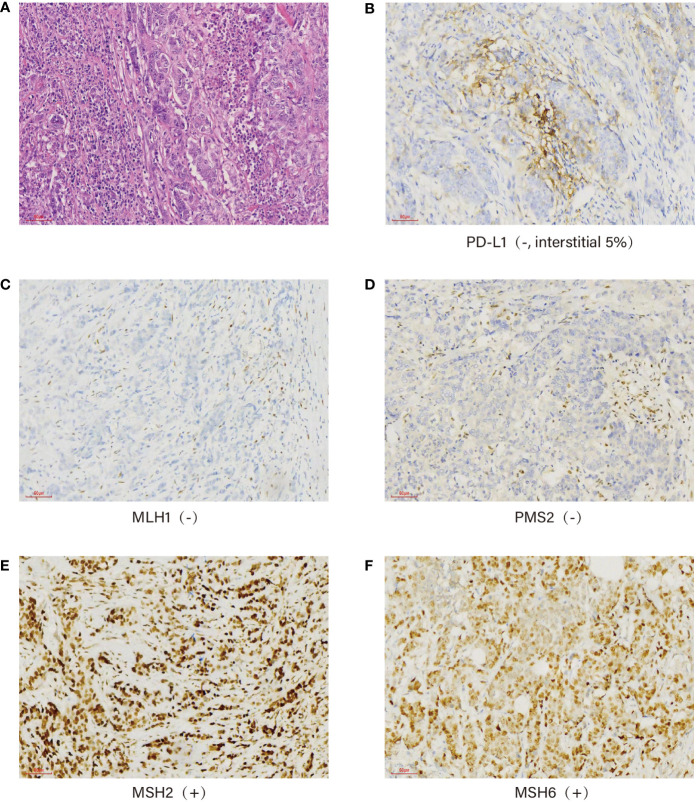
Immunohistochemistry: Pathological picture of the tumor confirmed under a ×40 microscope **(A)**, PD-L1 (-, interstitial 5%) **(B)**, mismatch repair protein expression: MLH1 (-) **(C)**, PMS2 (-) **(D)**, MSH2 (+) **(E)**, and MSH6 (+) **(F)**.

The postoperative pathological examination showed a “poorly differentiated adenocarcinoma (diffuse infiltrative type, 6 × 5 × 1.2 cm) on the side of the lesser curvature of the gastric sinus, invading the plasma membrane, pancreatic adhesions, cancerous thrombus in the ducts, invasion of nerves, lymph nodes of the lesser curvature (5/7), lymph nodes of the greater curvature (1/5), lymph nodes of “no. 7” (1/4), lymph nodes of “no. 8” (1/5), and in the “transverse colonic mesentery”. The “upper and lower margins”, the omentum, the “hepatic ligament”, and the “nos. 4, 6, and 12a lymph nodes” were negative for fibrofatty tissue. The immunohistochemistry results were as follows: CEA (+), P16 (++), Ki67 (80%), P53 (-), ER (-), PR (-), HER2 (-), PD-L1 (-, interstitial 5%), MLH1 (-), PMS2 (-), MSH2 (+), and MSH6 (+) (**Figures 2A–F**). According to the American Joint Committee on Cancer (AJCC) Cancer Staging Manual (Springer International Publishing, 8th Edition 2018), the tumor was classified as pT4bN3M0, stage III C.

**Figure 3:**
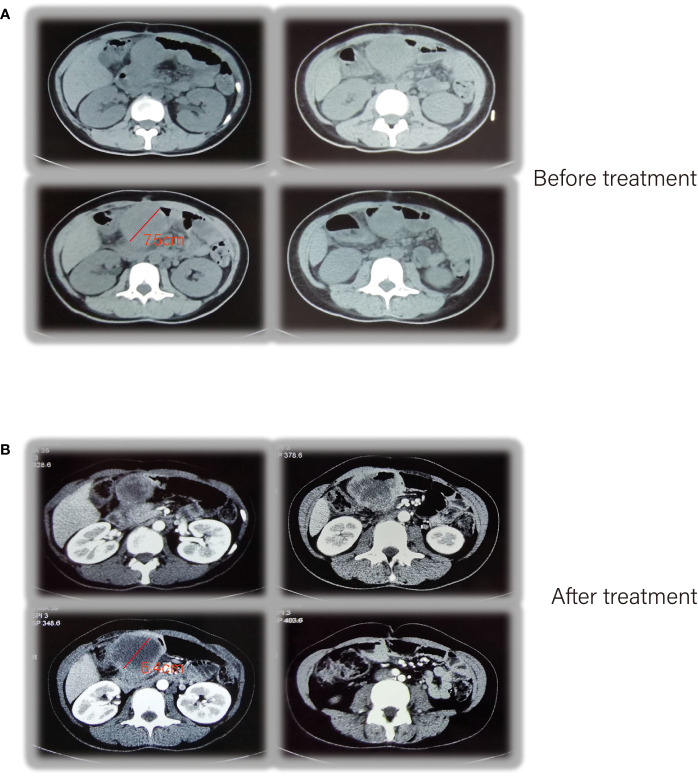
CT enhancement of the abdomen showed tumor recurrence **(A)**, after receiving pembrolizumab therapy **(B)**.

Unfortunately, postoperative follow-up at 1 year revealed tumor recurrence. CT enhancement of the abdomen showed a soft tissue mass measuring approximately 7.5 cm × 5.2 cm, considered as tumor recurrence/metastasis with a possible involvement of the duodenal stump, pancreatic head capsule, and adjacent transverse colon mesentery, with enlarged lymph nodes in the hilar region and retroperitoneum (**Figure 3A**). The PET-CT results similarly confirmed this result (**Supplementary Figure S2**). Due to the refusal of using any chemotherapy for personal reasons, the patient received a pembrolizumab injection of 100 mg (2 mg/kg Q3W) intravenous drip for the first time on September 3, 2019 (6–9). (In view of the scarcity of cases treated with PD-1 inhibitors alone, we referred to the relevant medication guidelines and empirically used the relevant doses).

After the treatment, abdominal CTA was performed and, a mass measuring approximately 6.4 cm × 6 cm was seen on the right side of the mid-upper abdomen, which was significantly enhanced in a circular pattern with non-enhancing ground-density necrotic foci and gas shadows within, with scattered calcifications visible in the posterior superior wall and disappearance of the fatty gap with the surrounding intestinal canal (**Figure 3B**).

**Figure 4:**
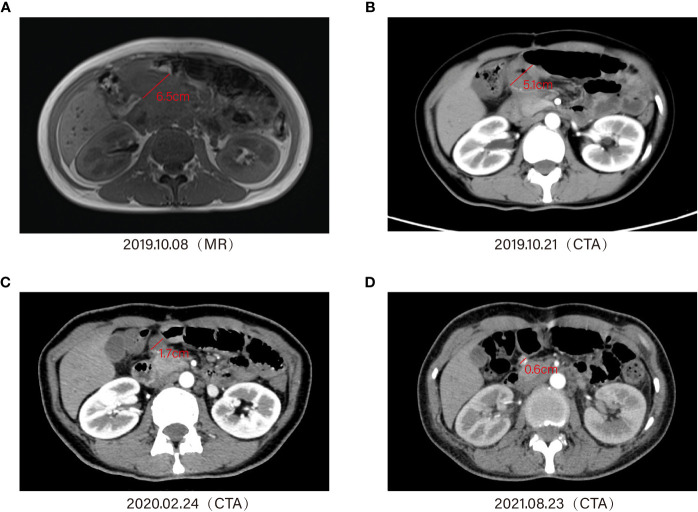
Timeline of treatment and diagnostic assessment about the patient who received a pembrolizumab injection of 100 mg, intravenous drip regularly. Magnetic resonance imaging (MRI) and enhanced computed tomography (CT) showed the lesion shrinking gradually **(A–D)**.

On the day of pembrolizumab administration, she suffered a high fever of 39°C –40°C with chills, cough, dyspnea, profuse sweating, malaise, hypotension (systolic blood pressure 70–60 mmHg), SpO_2_ 80%–90%, WBC 19.87 × 10^9^/L, and N% 80.3%, accompanied with normal hepatorenal function. Considering an infusion reaction or drug allergy to immunotherapy, aggressive symptomatic supportive therapy, physical hypothermia, oxygen inhalation, volume expansion, and rehydration were given. At 3 days later, the patient’s symptoms were significantly relieved, and the WBC (white blood cells) were also gradually decreasing in the routine blood tests. Then, on September 29, 2019, the patient received a pembrolizumab injection of 100 mg intravenous drip for the second time. On October 8, 2019, MRI enhancement of the abdomen suggested that the soft tissue mass in the right upper abdomen measured approximately 6.5 cm × 4.0 cm (**Figure 4A**). On October 19, 2019, the patient received a pembrolizumab injection of 100 mg intravenous drip for the third time. The CTA for the abdomen suggested a patchy shadow of the duodenum and peripancreatic head in the right upper abdomen, approximately 5.1 cm × 4.0 cm in diameter. The duodenum and peripancreatic head lesion was smaller than 19–10-8, multiple lymph nodes in the mesentery, hilar region, and retroperitoneum (**Figure 4B**). During the period November 11, 2019–February 24, 2020, the patient received a pembrolizumab injection of 100 mg intravenous drip regularly, and the lesion had shrunk to 1.7 cm in diameter (**Figure 4C**).

The patient developed complications such as rash and vitiligo during this period. In addition, during the last application of immunotherapy, the patient experienced sudden hypotension (85/42 mmHg) with oxygen saturation of 88.4% and was transferred to the surgical ICU ward of our hospital. In addition, the patient’s troponin level was 1.30 ng/ml, the myoglobin level was 11.60 ng/ml, and the creatine kinase isoenzymes were 15.8 ng/ml, indicating drug-responsive hypotension and myocarditis. After receiving norepinephrine, dobutamine, and glucocorticoids to boost the heart pressure and anti-inflammatory effects, the symptoms were improved and the patient was discharged. At subsequent follow-up, the whole abdomen CTA (August 23, 2021) suggested that the duodenal and peripancreatic head lesion reduced to 0.6 cm, with a bilateral ovarian pattern in full view and multiple lymph nodes in the mesentery, hilar region, and retroperitoneum (**Figure 4D**).

Subsequently, due to the patient’s resistance to treatment, she did not receive further immunotherapy. Until at follow-up in August 2023, except for being diagnosed with undifferentiated connective tissue disease (UCTD) in February 2023, there were no signs of tumor recurrence. During the follow-up, the corresponding tumor markers showed a gradual downward trend (**Supplementary Figure S3**).

## Discussion

The group of pregnancy-related gastric cancer patients has received considerable attention in recent years. In general, gastric cancer, perceived to have the fifth highest incidence of malignancies worldwide, is one of the four leading causes of cancer-related deaths (10). Actually, gastric cancer is the most commonplace cancer in the middle-aged and elderly population (average age of prevalence is 60 years old) and is less common in people under 40 years of age. Less than 15% of all gastric adenocarcinomas occur in adults younger than 41 years of age, and some scholars believed that the rare gastric cancer during pregnancy has the same features as in other patients under 40 years (11, 12).

Due to a great quantity of factors, the difficulty of diagnosing gastric cancer during pregnancy is significantly increased, leading to pregnant patients with gastric cancer being diagnosed at a progressive or even advanced stage, which gives birth to an extremely poor prognosis for survival (13–15). This has alerted the requirements to attach importance to gastrointestinal symptoms during pregnancy, which includes conducting timely and comprehensive physical examinations, checking for tumor markers, and performing fiber optic gastroscopy to exclude gastric lesions (16–18). For pregnant patients with gastric cancer, the timing of systemic chemotherapy intervention, surgical intervention to reduce the impact on the fetus during delivery, and treatment for the fetus during different trimesters are key areas for further research (19–21). According to previous experience, most of the patients reported in China with gastric cancer in pregnancy are treated with SOX regimen chemotherapy after surgery. The first-line chemotherapy regimens for patients with advanced disease are dominated by a combination of platinum- and fluorouracil-based regimens, and the second-line chemotherapy regimens contain irinotecan or raltitrexed (22).

Additionally, the results of several studies demonstrated that HER-2-positive patients with advanced gastric cancer will benefit from trastuzumab treatment. There are no significant differences in HER-2 expression and amplification between gastric cancer patients in pregnancy and the non-pregnant ones. Therefore, HER-2 testing should be routinely recommended for patients with gastric cancer during pregnancy (23). With the advent of the era of immunotherapy, immunotherapy combined with chemotherapy has gradually become the first-line treatment choice for advanced gastric cancer. The efficacy of immunotherapy in MSI-H patients is satisfactory (24–26).

Immune checkpoint blockade is now regarded as a strategy for chemorefractory gastric cancer. The phase 2 non-randomized KEYNOTE-059 trial resulted in improved overall survival for an international group of chemorefractory gastric cancer patients treated with pembrolizumab (27). Currently, more antibodies targeting PD-L1 have also become commercially available (28, 29). Novel pharmaceutics development like computational analysis of PD-L1 may be allowed for the accurate determination of antigen–antibody interactions, which could improve the efficiency of immunotherapy (30, 31). However, the clinical practice of anti-PD-1 monotherapy only involves some special cancer species, and the efficacy is still controversial (32, 33). There have been only very few reports suggesting the use of immune checkpoint inhibitors in pregnant patients with melanoma (34).

To date, there are a few reports of satisfactory outcomes with PD-1 inhibitors alone after surgery in pregnancy-related gastric cancer patients, and there are no precise clinical guidelines recommending the use of PD-1 inhibitors alone. Furthermore, relevant studies have shown that the use of immune checkpoint inhibitors leads to an increased incidence of spontaneous abortion, stillbirth, and preterm birth from the onset of fetal organ development to delivery (35, 36). To the best of our knowledge, we provide the first case report of a satisfactory outcome with immunotherapy alone in a pregnancy-related gastric cancer patient. This patient opted for the termination of pregnancy and radical surgery, given a variety of subjective or objective factors. This case also refused conventional chemotherapy and received only immunotherapy alone. As a matter of fact, the discovery of tumor recurrence after surgical treatment in this case poses a greater challenge to the clinical surgeon. How should the treatment after recurrence be chosen? How about the choice of chemotherapy regimen? Is there an opportunity to consider re-surgical intervention? Does the patient still have a requirement for fertility and will she consider *in vitro* fertilization–embryo transfer again? It is pleasing to note that immunotherapy alone has shown promising results in controlling the recurrent lesions during the subsequent follow-up. The favorable outcome of the pregnancy-related gastric cancer patient treated with PD-1 inhibitors alone may be related to the alteration of the immune microenvironment by pregnancy-related hormones. Moreover, some related reports have yet to mention a novel mechanism in trophoblasts to create a tolerant fetal–maternal interface by upregulating PD-L1. Tumors may also use PD-L1 expression to evade the host’s immune response, thereby promoting their survival. Moreover, it has been hypothesized that age-related changes to the immune system may impact the efficacy and toxicity of immunotherapy. Nevertheless, there have been few studies in younger adults (e.g., under 40 years). Thus, it remains unclear whether immunotherapy has a different efficacy in this subgroup (37).Therefore, the application of PD-1 inhibitors may partially serve to disarm immune escape and strengthen anti-tumor immunity in gravida, but the exact mechanism needs to be further investigated (38).

In conclusion, the diagnosis and treatment of a pregnancy-related gastric cancer patient need to be balanced with multiple factors. This case report has provided some treatment options for related cases.

## Conclusions

The treatment of pregnancy-related gastric cancer is quite tricky in clinical practice.

In this case report, we focus on a 32-year-old pregnant patient diagnosed with gastric cancer. Radical surgery was conducted after the termination of pregnancy. At 1-year postoperative follow-up, tumor recurrence was shown. Fortunately, this patient has achieved a quite favorable outcome after having received anti-PD-1 alone. For similar cases, questions such as when to intervene with systemic chemotherapy in pregnant patients with gastric cancer, when to perform surgical intervention with minimal impact on fetal delivery, how to weigh the timing of fetal treatments in different trimesters, and whether single-agent immunotherapy results in favorable outcomes deserved to be studied in depth. This case report provides some treatment options for related cases.

## Data availability statement

The raw data supporting the conclusions of this article will be made available by the authors, without undue reservation.

## Ethics statement

Written informed consent was obtained from the individual(s) for the publication of any potentially identifiable images or data included in this article.

## Author contributions

XuL: Writing – original draft, Writing – review & editing. XiL: Writing – review & editing. CZ: Writing – review & editing. LJ: Writing – review & editing.

## Supplementary material

Supplementary Figure 1 Electron fiber optic gastroscopy: large, ulcerated lesion of the gastric sinus, involving the four walls and the gastric angle, and the lesser curvature of the gastric body.

Supplementary Figure 2 PET-CT detected a tumor recurrence in the right upper abdomen.

Supplementary Figure 3 CA724, CA199, and CA125 decreased gradually during follow-up.
