# Supplementary figures and images for: Effective control of postoperative recurrence of pregnancy-related gastric cancer using anti-PD-1 as a monotherapy: a case report
Source: Front Oncol. 2024 May 10;14:1321149. doi: 10.3389/fonc.2024.1321149 (PMC11116784; doi:10.3389/fonc.2024.1321149)

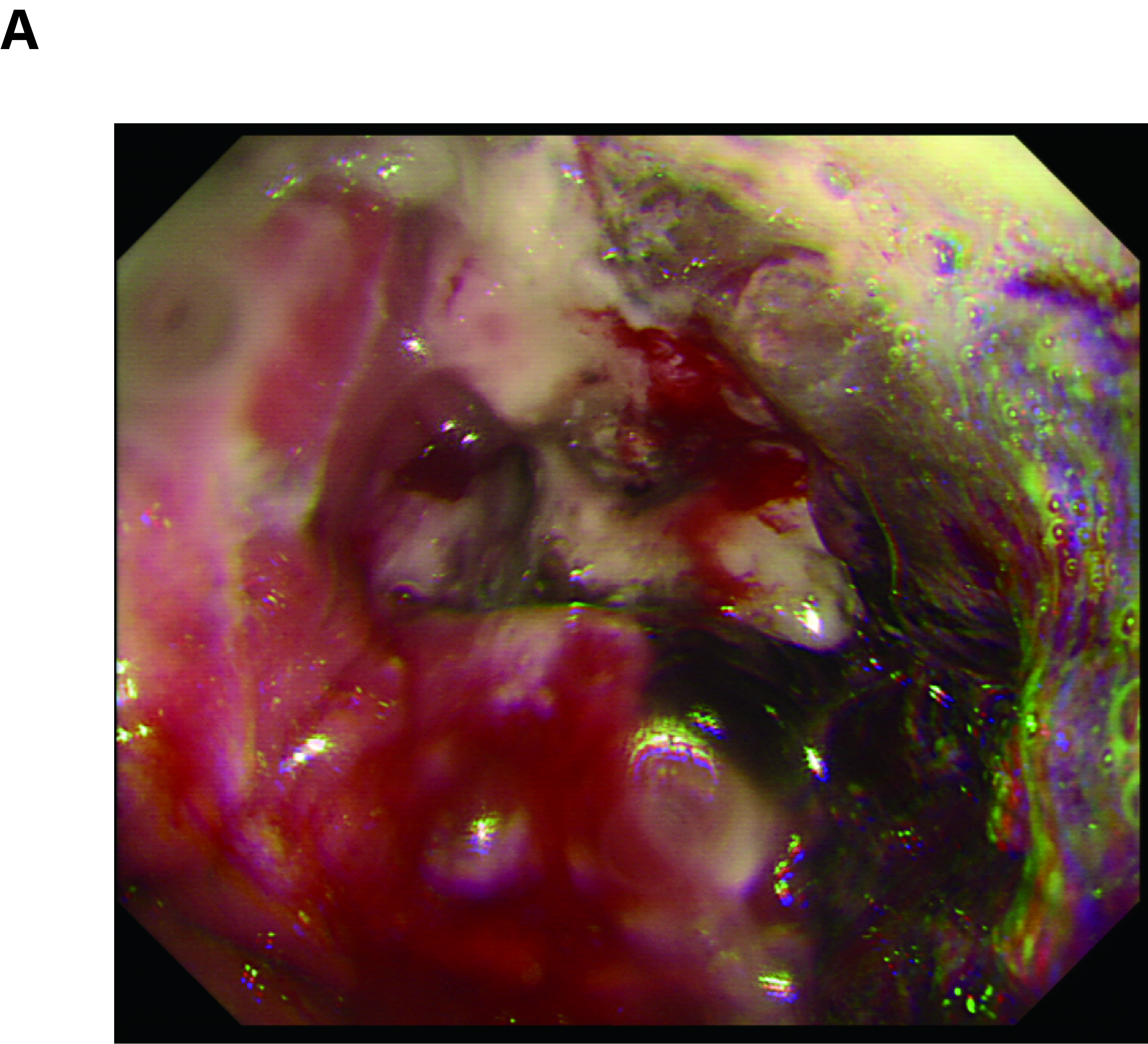

Supplement: Supplementary Figure 1 — Electron fiber optic gastroscopy: large, ulcerated lesion of the gastric sinus, involving the four walls and the gastric angle, and the lesser curvature of the gastric body. [file Image_1.tif]

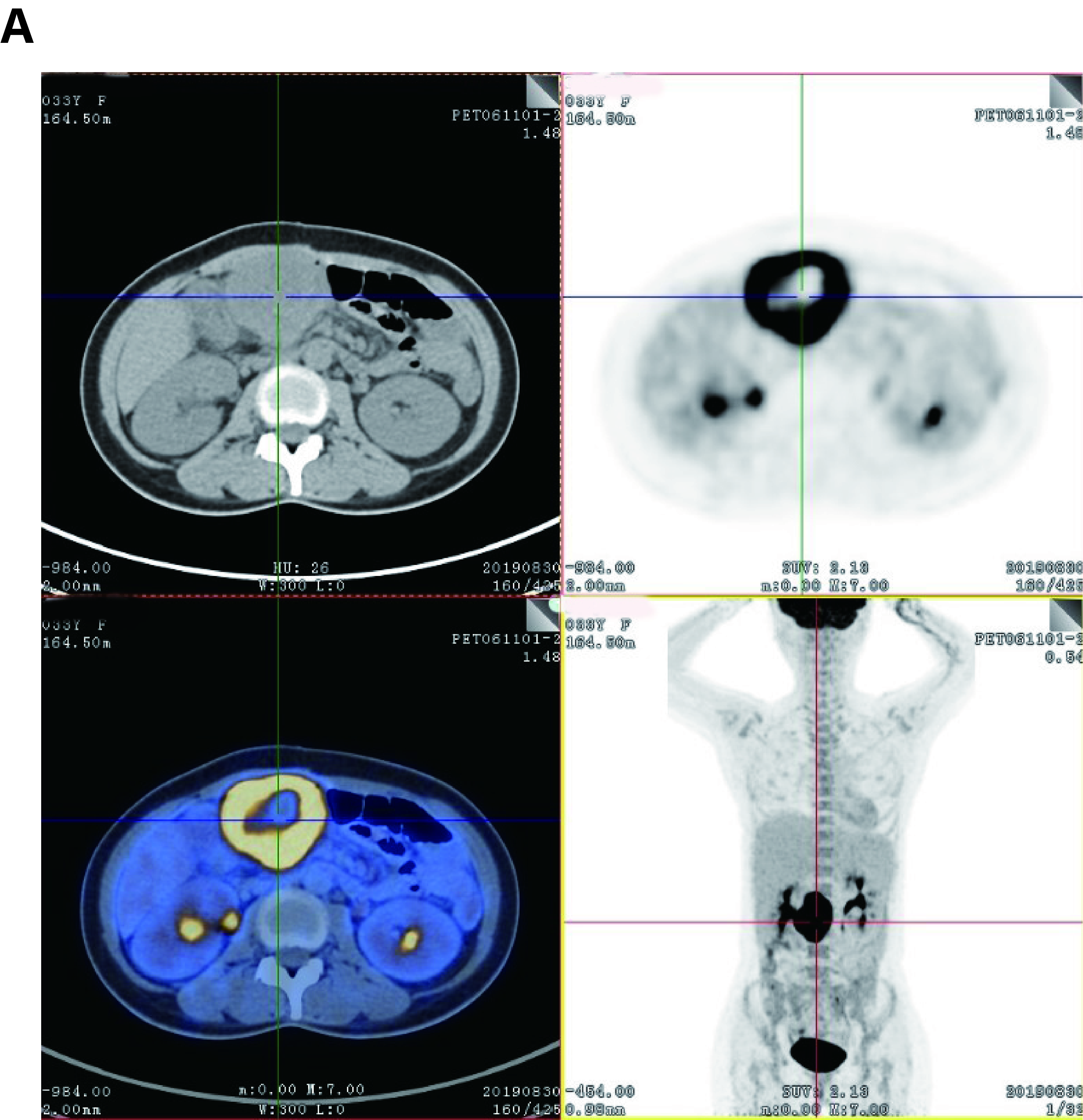

Supplement: Supplementary Figure 2 — PET-CT detected a tumor recurrence in the right upper abdomen. [file Image_2.tif]

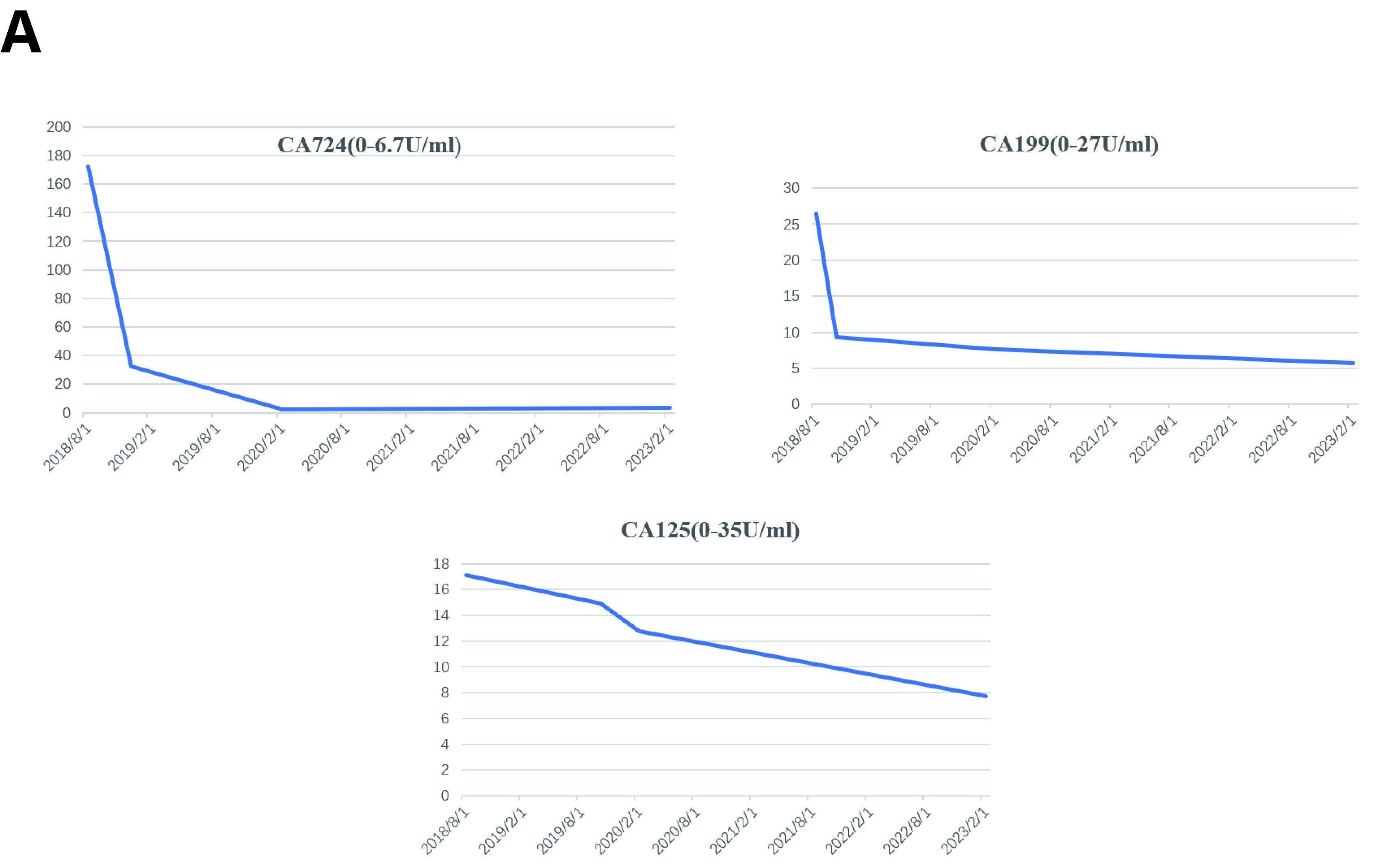

Supplement: Supplementary Figure 3 — CA724, CA199, and CA125 decreased gradually during follow-up. [file Image_3.tif]
